# Supplementary material for: Cost analysis of photobiomodulation in tibia fracture in the Brazilian public health system
Source: PLoS One. 2023 Dec 8;18(12):e0294290. doi: 10.1371/journal.pone.0294290 (PMC10707925; doi:10.1371/journal.pone.0294290)
Supplement: Attachment — Submitted filename: Review Cost Analysis Tibia Plos One.docx [file pone.0294290.s002.docx]

This manuscript has a lot of potential to be a top-tier publication and the subject area is very relevant. Unfortunately, the work has been poorly conducted. The methods and the result section need to be re-arranged and done to meet the minimum health economic standards see CHEERS checklist.

Background/ Introduction

- There is more emphasis on the UK and US rather than Brazil where the research was conducted. I would suggest taking a more global approach. There a quite a number of systematic reviews that are related to this that can be cited.
- Include a paragraph on economic evaluation/analysis relevant to the subject matter in the Background section.

Methods

- In health economic analysis the term “cost-effectiveness” applies to the “Incremental cost-effectiveness ratio (ICER)”, in this study “cost-effectiveness” is being misused to mean “cost per effect”.
- What are the characteristics or demographics of the sample population (i.e. the 27 participants)? This should be the first table.
- The study did not mention the intervention cost. The intervention cost is what differentiates the intervention and control group costs. (See for example Cost-effectiveness Analysis of the Dental RECUR Pragmatic Randomized Controlled Trial: Evaluating a Goal-oriented Talking Intervention to Prevent Reoccurrence of Dental Caries in Children, <https://link.springer.com/article/10.1007/s40258-022-00720-5>)

Results

- Table 1 represents the cost, is there a reference to the costs or are the hypothetical?
- Tables 2, 3, 4, and 5 can be combined together (see table 4 of example paper: Cost-effectiveness of Memory Assessment Services for the diagnosis and early support of patients with dementia in England, <https://journals.sagepub.com/doi/full/10.1177/1355819617714816>; and table 3 of example paper: Cost-Effectiveness of Dementia Care Mapping in Care-Home Settings: Evaluation of a Randomised Controlled Trial, <https://link.springer.com/article/10.1007/s40258-019-00531-1>).
- The term Effectiveness in the third column of Table 2 should be labelled “Effect”
- No need to include formulas in the table. The methods section describes and references all types of formulas and methods.
- Tables 6 and 7 can be combined if at all it needs to be there.

Discussion

- The discussion will improve after the methods and result section are updated.
