# Supplementary material for: Cost analysis of photobiomodulation in tibia fracture in the Brazilian public health system
Source: PLoS One. 2023 Dec 8;18(12):e0294290. doi: 10.1371/journal.pone.0294290 (PMC10707925; doi:10.1371/journal.pone.0294290)
Supplement: Attachment — Submitted filename: Review PONE -D-23-17845.docx [file pone.0294290.s003.docx]

Dear authors! This investigated topic is very important, however, I have some questions that were not clear for my understanding, for example, was the control group matched? I saw that there were 27 participants in all. Did the inclusion criteria include similar ages and systemic health conditions? It would be interesting if in the abstract, methodology, these data were clarified for the reader. Likewise, in the discussion of the study, these data were presented and discussed, as it is known that both bone repair and soft tissue wounds are impacted by systemic conditions.

Another aspect I would like to point out is that the BATES-JENSEN scale should be presented to the reader, bearing in mind that this scale was the main criterion for defining the effectiveness of photobiomodulation therapy. Although previous work has been published, I think it is important to describe the photobiomodulation protocol in detail.

Reading the work, the question of the cost of the professional and the assistant team came up. Was it not possible to include this data? If it was not possible, wouldn't it be interesting to also discuss this aspect as a limitation of the study? I believe that only hospital costs were presented, but we know that there are often extra costs borne by patients. It would be interesting, therefore, to add this information.
