# Supplementary material for: Cost analysis of photobiomodulation in tibia fracture in the Brazilian public health system
Source: PLoS One. 2023 Dec 8;18(12):e0294290. doi: 10.1371/journal.pone.0294290 (PMC10707925; doi:10.1371/journal.pone.0294290)
Supplement: Attachment — Submitted filename: Response_letter.docx [file pone.0294290.s004.docx]

**Response letter**

**Dear Editor**

**Melissa Orlandin Premaor**

Initially, we thank you for taking the time to evaluate the article and for considering it for publication.

We have reviewed the points suggested by the reviewers.

The changes are in yellow in the text, and each point was answered for each reviewer.

Based on the evaluations received, we present below the changes made and their justifications:

**Reviewer #1**: *This manuscript has a lot of potential to be a top-tier publication and the subject area is very relevant. Unfortunately, the work has been poorly conducted. The methods and the result section need to be re-arranged and done to meet the minimum health economic standards see CHEERS checklist.*

**Response**: We appreciate your observation, and to improve the description in the methods and results sections, we followed the CHEERS checklist and adjusted the information in the article. Additionally, we have attached the checklist as supplementary material.

**Background/ Introduction**

*There is more emphasis on the UK and US rather than Brazil where the research was conducted. I would suggest taking a more global approach. There a quite a few systematic reviews that are related to this that can be cited.*

*Include a paragraph on economic evaluation/analysis relevant to the subject matter in the Background section*.

**Response:** We understand your perspective and agree. We have added more cost details and information from the systematic review by Schade A, Khatri C, Nwankwo H, Injury WC, 2021 undefined. The economic burden of open tibia fractures: A systematic review. Elsevier [Internet]. [cited 2022 Sep 1]; Available from: https://www.sciencedirect.com/science/article/pii/S002013832100125X**.**

**Methods**

*In health economic analysis the term “cost-effectiveness” applies to the “Incremental cost-effectiveness ratio (ICER)”, in this study “cost-effectiveness” is being misused to mean “cost per effect”.*

*What are the characteristics or demographics of the sample population (i.e. the 27 participants)? This should be the first table.*

*The study did not mention the intervention cost. The intervention cost is what differentiates the intervention and control group costs. (See for example Cost-effectiveness Analysis of the Dental RECUR Pragmatic Randomized Controlled Trial: Evaluating a Goal-oriented Talking Intervention to Prevent Reoccurrence of Dental Caries in Children, https://link.springer.com/article/10.1007/s40258-022-00720-5)*

**Response**: We appreciate the suggestion to adjust the terms. We have made the necessary changes, replacing the term "effectiveness" with "effect," as suggested. Regarding the cost of the intervention, as this is the first clinical trial conducted with photobiomodulation in this clinical condition, we do not have data on the cost of the procedure in the Brazilian healthcare system. Therefore, following the methodology of health economic analysis, we considered the cost of the equipment as the cost of the intervention since the only difference between the PBM and sham groups was the application of light. The equipment costs were added to the costs of the experimental group. We have made a modification in the article's wording to clarify this point.

We have included the demographic information in the text and emphasized the reference to the clinical trial that presents the table of original clinical data (Jana Neto FC, Martimbianco ALC, Mesquita-Ferrari RA, Bussadori SK, Alves GP, Almeida PVD, et al. Effects of multiwavelength photobiomodulation for the treatment of traumatic soft tissue injuries associated with bone fractures: A double-blind, randomized controlled clinical trial. J Biophotonics. 2023 May 1;16(5). https://onlinelibrary.wiley.com/doi/10.1002/jbio.202200299)

**Results**

*Table 1 represents the cost, is there a reference to the costs or are the hypothetical?*

*Tables 2, 3, 4, and 5 can be combined together (see table 4 of example paper: Cost-effectiveness of Memory Assessment Services for the diagnosis and early support of patients with dementia in England, https://journals.sagepub.com/doi/full/10.1177/1355819617714816; and table 3 of example paper: Cost-Effectiveness of Dementia Care Mapping in Care-Home Settings: Evaluation of a Randomised Controlled Trial, https://link.springer.com/article/10.1007/s40258-019-00531-1).*

*The term Effectiveness in the third column of Table 2 should be labelled “Effect”*

*No need to include formulas in the table. The methods section describes and references all types of formulas and methods.*

*Tables 6 and 7 can be combined if at all it needs to be there.*

**Response**: We agree with the reviewer's observation and, as suggested, we have removed the formulas and have left only the explanation of the analysis in the methodology section. We have grouped the data in the tables to make it clearer for the reader.

**Discussion**

*The discussion will improve after the methods and result section are updated.*

**Response**: We sought to improve the discussion following the new corrections and inclusions.

**Reviewer #2:** *Dear authors! This investigated topic is very important, however, I have some questions that were not clear for my understanding, for example, was the control group matched? I saw that there were 27 participants in all. Did the inclusion criteria include similar ages and systemic health conditions? It would be interesting if in the abstract, methodology, these data were clarified for the reader. Likewise, in the discussion of the study, these data were presented and discussed, as it is known that both bone repair and soft tissue wounds are impacted by systemic conditions.*

**Response**: Thank you for the feedback. To clarify the doubts and improve the description in the text of the studied sample, we have added details about the sample and the studied groups. The 27 participants were randomly divided into 2 study groups. One group received photobiomodulation therapy with 13 participants, while the group referred to as "sham" received the same procedures as the experimental group, but with the photobiomodulation equipment turned off. We have included the inclusion and exclusion criteria in the methods description. As the demographic data of the sample has already been published along with the clinical data, we have included this information in the text. We hope to have addressed this deficiency in the methodological description.

*Another aspect I would like to point out is that the BATES-JENSEN scale should be presented to the reader, bearing in mind that this scale was the main criterion for defining the effectiveness of photobiomodulation therapy. Although previous work has been published, I think it is important to describe the photobiomodulation protocol in detail.*

**Response**: We agree that the BATES-JANSEN scale is one of the main points of our assessment. Considering the observation, we have highlighted the description of the scale and its scoring in the methodology section. In the results section, we have also provided sample results based on the BATES-JENSEN scale. Table 2 contains the data we deem essential for conducting the economic analysis.

*Reading the work, the question of the cost of the professional and the assistant team came up. Was it not possible to include this data? If it was not possible, wouldn't it be interesting to also discuss this aspect as a limitation of the study? I believe that only hospital costs were presented, but we know that there are often extra costs borne by patients. It would be interesting, therefore, to add this information.*

**Response**: We agree with the reviewer that the costs of the professional team and patient-related costs are of utmost importance in economic evaluations; however, this is an initial study. It is not yet a procedure included in the Brazilian healthcare system's list of procedures, and therefore, it was not possible to gather this data. But we believe that this preliminary economic evaluation provides us with a foundation for developing the methodology for the next clinical trial, which will include assistant team costs and indirect costs in this treatment context. We have included this information as a study limitation.
