# Supplementary material for: Cost analysis of photobiomodulation in tibia fracture in the Brazilian public health system
Source: PLoS One. 2023 Dec 8;18(12):e0294290. doi: 10.1371/journal.pone.0294290 (PMC10707925; doi:10.1371/journal.pone.0294290)
Supplement: Attachment — Submitted filename: PlosOne Review v2.docx [file pone.0294290.s005.docx]

Table 3: The first column with the Ratio should differ from the second column with the Ratio. The second column ratio should be indicated as ICER (Incremental cost-effectiveness ratio).

In the last row last column of Table 3, what does (negative) mean?? This is ambiguous and should be taken out.

In the text the word “negative” should be removed e.g “The cost-effectiveness ratio compared to the two groups was R$3,500.98 (negative)”. This sentence does not support or negate any intervention, does it mean the PMB group was better, or was the control better? All economic evaluations are comparisons of at least two interventions. This word negative was used in 6 (six) other places, can they be removed?
