# Supplementary material for: Cost analysis of photobiomodulation in tibia fracture in the Brazilian public health system
Source: PLoS One. 2023 Dec 8;18(12):e0294290. doi: 10.1371/journal.pone.0294290 (PMC10707925; doi:10.1371/journal.pone.0294290)
Supplement: Attachment — Submitted filename: response_reviewer_plos_13_10.docx [file pone.0294290.s006.docx]

Dear Reviewers,

First and foremost, we would like to express our sincere gratitude for your invaluable time. Please find below the responses to your queries and suggestions:

***Reviewer #1:*** ***Table 3: The first column with the Ratio should differ from the second column with the Ratio. The second column ratio should be indicated as ICER (Incremental cost-effectiveness ratio).***

***In the last row last column of Table 3, what does (negative) mean?? This is ambiguous and should be taken out.***

***In the text the word “negative” should be removed e.g “The cost-effectiveness ratio compared to the two groups was R$3,500.98 (negative)”. This sentence does not support or negate any intervention, does it mean the PMB group was better, or was the control better? All economic evaluations are comparisons of at least two interventions. This word negative was used in 6 (six) other places, can they be removed?***

**Response to Reviewer:** We appreciate the suggestion for the correction in Table 3 regarding the columns of ratio and ICER. We have made the correction to the second column of ratio as suggested.

We agree that the word "negative" may introduce difficulties in comprehension of the analysis. We have removed it from Table 3 and all other instances where it appeared.

By removing the word "negative," we reformulated the text to explain that the term "negative" signifies a savings of R$3,500.98 when employing the PBM technique. Thus, in our analysis, PBM outperformed the control group.

We've marked them in yellow for your convenience. Additionally, you can also find the changes outlined below:

The cost-effectiveness index between the two groups was R$3,500.98, indicating a savings of R$3,500.98 with the PBM technique. In addition to the observed difference in the clinical trial results, considering this simplified analysis, the PBM group demonstrated greater cost-effectiveness, spending less with a lower daily average on the scale. Table 4 illustrates the model used for the calculation of cost-effectiveness. The proposed intervention did not incur additional costs, as the disparity in costs for reducing measurements between the groups was smaller for the PBM group.

The ICER for this outcome represented a savings of R$752.71 for every ten days per resolved wound. It is assumed that if the entire sample had been treated with PBM, it would be possible to save R$7,527.10 and reduce the resolution time by ten days compared to the Control group (Table 3).

***Reviewer #2: Dear authors, I think that the work has now been improved with the information that was suggested. As you highlighted that it is a preliminary study, my suggestion is that you continue to investigate the cost-benefit and effectiveness of this treatment modality and that it can actually be incorporated into the public health system of different countries. Mainly from countries that require scientific evidence for the incorporation of therapeutic protocols and/or new healthcare technologies.***

**Response to Reviewer:** We sincerely appreciate your valuable suggestion. Indeed, it is highly pertinent. We will certainly continue our investigations into the cost-benefit and effectiveness of this treatment modality. We are committed to assessing its feasibility for integration into the public health system. Thank you for your insightful guidance.

We appreciate your valuable time to evaluate our work and your valuable suggestions.

We hope the article aligns with your expectations with the changes made.

Thank you once again for your consideration.

Sincerely,

Authors
